# Evaluation of a collaborative care approach between general practitioners and clinical pharmacists in primary care community settings in elderly patients on polypharmacy in Slovenia: a cohort retrospective study reveals positive evidence for implementation

**DOI:** 10.1186/s12913-019-3942-3

**Published:** 2019-02-13

**Authors:** Matej Stuhec, Katja Gorenc, Erika Zelko

**Affiliations:** 1 Department of Clinical Pharmacy, Ormoz Psychiatric Hospital, Ptujska cesta 33, SI-2270 Ormoz, Slovenia; 2 Faculty of Pharmacy, University of Ljubljana, Askerceva cesta 7, SI-1000 Ljubljana, Slovenia; 3 Faculty of medicine Maribor, University of Maribor, Taborska ulica 8, 2000 Maribor, Slovenia

**Keywords:** Clinical pharmacist, Primary care, General practitioners, Service implementation, Healthcare system, Polypharmacy, Elderly

## Abstract

**Background:**

The population of developed countries is aging, leading to an increase in the use of medication in daily practice, which can lead to serious treatment costs and irrational polypharmacy. A collaborative care approach, such as providing medication review service provided by a clinical pharmacist (CP), is a possible way to reduce drug-related problems and irrational polypharmacy. The aim of this study was to determinate whether a CP’s medication review service can improve the quality of drug prescribing in elderly patients treated with polypharmacy in primary care.

**Methods:**

In a retrospective observational medical chart review study, patients aged 65 years or more in the period 2012–2014 who received 10 or more medications concomitantly and who were screened by a CP were included. Data on pharmacotherapy and CPs’ interventions were obtained from the patients’ medical records (non-electronic chart review). Potential drug-drug interactions (pDDIs) were determined with Lexicomp Online™ 3.0.2. Only potential X-type DDIs (pXDDIs) were included. Potentially inappropriate medications in the elderly (PIMs) were identified using the PRICUS list.

**Results:**

Ninety-one patients were included. The CPs suggested 625 interventions, of which 304 (48.6%) were accepted by the general practitioners (GPs). After adopting the CPs’ interventions, the number of total medications decreased by 11.2% (*p* <  0.05) and the number of pXDDIs decreased by 42% (*p* <  0.05). The number of clinically important pXDDIs decreased by 50% (3 cases). The number of prescribed PIMs decreased by 20% (*p* = 0.069). The acceptance of CP’s recommendations reduced the number of pXDDIs (*p* < 0.05) and improved the adherence to heart failure treatment guidelines.

**Conclusions:**

A collaborative care approach offering a CP medication review service significantly improved the quality of pharmacotherapy by reducing the total number of medications and pXDDIs. The results support the implementation of this service in the Slovenian healthcare system.

## Background

The aging of the population in developed countries is accompanied by increasing medication and polypharmacy use [1]. According to the paper published by Maher et al., approximately 50% of older adults (aged > 65 years) took one or more medications that were not medically necessary [2]. Polypharmacy, defined as the use of multiple drugs or more than are medically necessary, often has negative consequences [3]. In a clinical setting, it is often connected with serious drug-drug interactions (DDIs) and potentially inappropriate medications (PIMs) in the elderly, which are an important cause of adverse events and increase morbidity, hospitalization rates, and total healthcare costs. In an Austrian cross-sectional study which included 48 out of 50 nursing homes in Austria, the authors found a high prevalence of PIMs. The prevalence of residents with at least one PIM was 70.3% (95% CI 67.2–73.4) [4, 5]. Inappropriate polypharmacy is wide-spread and potentially harmful and therefore requires prompt interventions [1, 6–8]. In addition, the literature review including 5 papers showed that polypharmacy continues to increase and its use was shown as a known risk factor for increased morbidity and mortality, which shows that health care professionals should check patients’ pharmacotherapy at each occasion [6].

Different approaches are possible to manage these problems. One of the newest is a collaborative care approach in which clinical pharmacists (CPs) are included in the management of patients’ pharmacotherapy (either as dependent or independent prescribers or a non-prescribers) [1]. Many studies found that including a pharmacist as a full member of the care team was associated with a substantially lower rate of adverse drug effects and medical errors [9–12]. In addition, the last Cochrane review on this topic suggested that non-medical prescribing by CPs and nurses was as effective as usual care medical prescribing, although effect sizes were small (non-medical prescribing was undertaken by nurses in 26 studies and by CPs in 20 studies). According to the results of this paper, the main benefits were seen in various cardiovascular treatments, patient satisfaction, and quality of life [13].

A collaborative care approach that includes CPs has been seen in some countries in which funders (e.g. national insurance companies) supported this service. In Slovenia, the support for collaborative care is due to a large increase in drug consumption in the last decade [14].

Although this system has been well described in the US and UK, there is little data on this topic in Central European countries, primarily because, by convention, the roles of pharmacists and physicians in these countries are rigidly separate [15]. Hence, this study aims to evaluate aspects of the completely new pharmaceutical service in Slovenia by examining whether medical reviews performed by CPs can improve the quality of drug prescribing in elderly patients treated with polypharmacy, measured in terms of better treatment guidelines adherence, the number of PIMs and the number of potential X-type drug-drug interactions (pXDDIs), major interactions that should be avoided.

## Methods

### Design and setting

This retrospective cohort study used paper medication reviews and medical charts, gathered from elderly patients (≥ 65 years), who were treated under the pilot trial *Pharmacist Consultant (non-prescriber)* at the Ljutomer Health Center, which caters to approximately 20.000 users in the northeast of Slovenia. The study was approved by the National Medical Ethics Committee of the Republic of Slovenia in 2016 (number = 0120–528/2016–2).

### Participants and data collection

Patients for this study were included from the pilot trial *Pharmacist Consultant (non-prescriber)* in Slovenia, which was funded by the Health Insurance Institute of Slovenia (ZZZS) between 2012 and 2015. In this pilot trial, a clinical pharmacy specialist (CP) was included into the general practitioners’ teams. Each CP was trained to perform a medication review and was a CP (a board-certified CP). All CPs in this pilot trial worked in the different hospital settings (e.g. psychiatric and general hospitals) and therefore they received their experiences on the hospital wards. Each team consisted of all only general practitioners (GPs). The CPs were based in primary community health centres (GPs’ offices). On the request of a GP, a patient could be referred to the CP for a medication review. After a consultation with the patient, the CP prepared a medication review that included potential drug-drug interactions (pDDIs), as identified by the Lexicomp Online™ software, possible adverse events, existing drug indications, potentially inappropriate medication in the elderly, an evaluation of drug adherence (refill-based system) and final recommendations depending on the patient’s outcomes. CPs mostly recommended drug discontinuation, drug initiation, dose adjustments and modifications of drug administration. GPs selected patients for referral to the CPs with a referral paper. The medication review was sent back to the GPs within a few days of the patient’s visit to the CP and the GPs could accept or reject their recommendations at the patient’s next regular visit. CPs communicated with GPs through the medication review and by phone call if necessary. On average, a CP produced 4–6 medication reviews in 6–8 h after a successful trial, this service has been adopted into the Slovenian healthcare system in 2016 [15].

The medication reviews and medical charts of the patients from the pilot trial *Pharmacist Consultant (non-prescriber)* were the source material for our retrospective study. The inclusion criteria for this study were the patients’ age (≥ 65 years), treatment at the Ljutomer Health Center, their referral to the CP in the period from 1.1.2012 to 31.12.2014, and concomittant use of 10 or more medications.

Over-the-counter medications, eye drops, and various dermal medications were excluded because it was often impossible to identify the manner of their use from the patients’ medical charts and medication reviews. Data on diagnoses, patient pharmacotherapy and CPs’ interventions were obtained from the patients’ paper medical records (medical charts) and paper medication reviews. The pDDIs were differentiated by interaction classes using the software Lexicomp Online™ 3.0.2 (free program), described and used in many previous trials [14, 16, 17]. Only pXDDIs were included in the final analysis, based on previous work by other another study [17]. Data on the clinical relevance of the pXDDIs were obtained from paper medication reviews, in which the CP recorded any adverse events caused by pXDDIs. Clinical data were recorded for all CP interventions. Pharmacotherapy details (e.g. medications, dose, pXDDIs) were also obtained from the medication reviews. Acceptance of recommendations was obtained from the patients’ charts at the first GPs’ visit. The study only included three CPs’ interventions: medication discontinuation, medication initiation and dose adjustment. Other interventions (e.g. administration instructions) were excluded. A medication review was performed according to the standard process, which has been already described above [15]. The PRISCUS list was used to identify potentially inappropriate medications for elderly patients (PIM) [18].

The data was compiled by a MPharm student (KG) under the supervision of a clinical pharmacist specialist (MS) directly from the patients’ paper medical charts and paper medication reviews. pXDDIs were collected by KG from paper medication reviews, because each medical review included pXDDIs and their categorization according to the Lexicomp Online™ software. The clinical relevance of pXDDIs was assessed by KG and MS directly from medication reviews if specified by a CP in the original medication review. The researchers did not contact any of the included patients during the data gathering process. Only Lexicomp Online™ was used in this study, because only this software was used in the pilot trial *Pharmacist Consultant*. A multivariable regression model that defines predictive factors for the impact of pXDDIs was conducted.

### Analysis

The baseline characteristics of patients were described as the mean ± standard deviation (SD) or median. Descriptive results were presented in graph form (number of PIMs according to the PRISCUS list before and after; total number of medications per patient before and after review; total number of pXDDIs before and after review). Total CP acceptance percentage by GPs (all recommendations) was calculated based on the three different CPs’ interventions: 1) medication discontinuation, 2) medication initiation, 3) dose adjustment. Analyses were carried out with the Statistical Package for Social Science 22.0 for Windows® (SPSS). A multivariable regression model (number of pXDDIs as a dependent variable) was developed with several independent variables (age, gender, total number of medications and clinical pharmacist acceptance), which were selected according to the important variables observed from the patients’ charts and medication reviews. In addition, heart failure treatment guidelines (European Society of Cardiology (ESC) Guidelines for the diagnosis and treatment of acute and chronic heart failure) were applied to evaluate the CPs’ adherence to these guidelines. For this purpose, the data of all patients with a diagnosis of heart failure was used [19].

## Results

### General data and clinical pharmacists recommendations

**Fig. 1:**
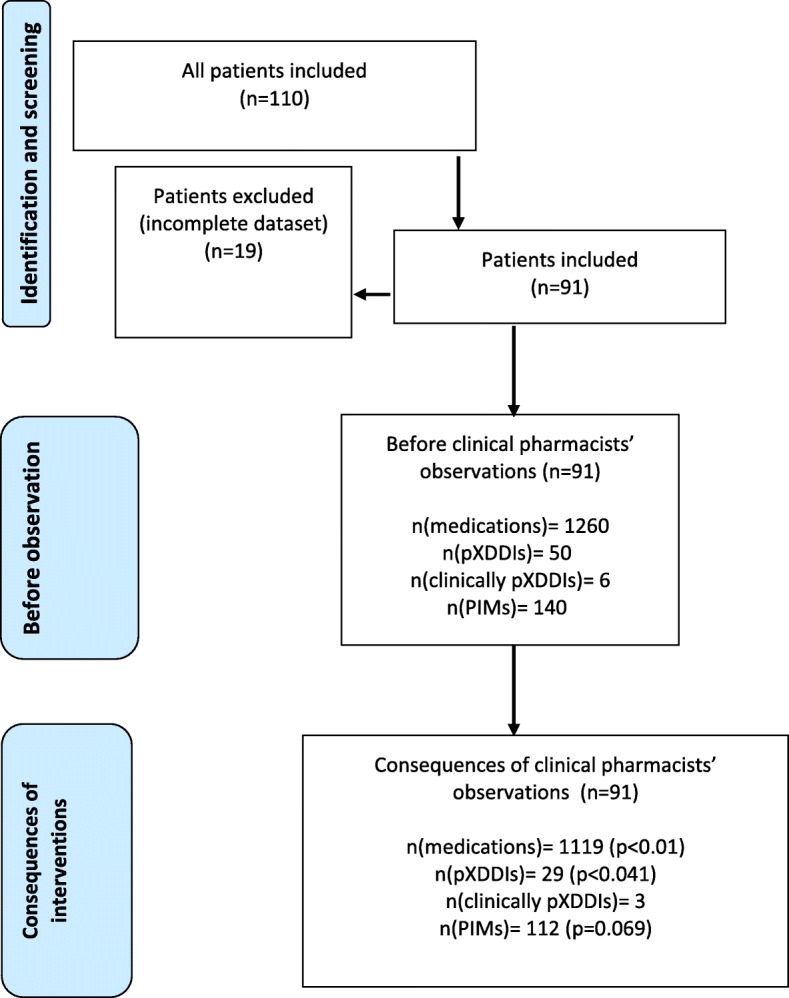
A flow chart of main study outcomes

Ninety-one patients were included in this study. Researchers excluded 19 patients before the study, because of incomplete datasets (e.g. missing variables, death patients without available medical charts). The study involved 56 women (61.5%) and 35 men (38.5%). The average age was 77.5 years (median 78, range 65–91). In total, patients received 1260 medications (mean 13.8, median 13) (regular therapy and therapy as needed) during the study. The highest number of prescribed medications was 21 per patient. The numbers of proposed CP’s interventions and GPs acceptances during the study are shown in Table 1. The CP proposed 625 interventions (median seven interventions per patient). The CP most often proposed seven different interventions per patient, which was the case in 103 (16.5%) patients. GPs accepted almost half of the proposed interventions (*n* = 304; 48.6%). Detailed results are presented in the Fig. 1 (flow chart).

**Table 1:** The numbers of interventions proposed and interventions accepted by general practitioners during the study

Proposed interventions	Total
Number of proposed interventions by clinical pharmacists	625
Number of interventions accepted by general practitioners	304
Median of the proposed interventions	7
Median accepted interventions	3
Maximum number of proposed interventions by clinical pharmacists	15
Maximum number of accepted interventions by general practitioners	8

At the end of the study, patients received 1119 medications in total (mean 12.3, median 12), which is 141 fewer than they received before the CP’s review (total number of prescribed medications decreased by 11.2%). Patients, whose GPs accepted the CPs’ recommendations, overall had fewer medications per patient compared to those whose GPs did not accept the recommendations (Mann-Whitney’s U test; U = 68,000; *p* < 0.01).

### Potential and clinically important drug-drug interactions and potentially inappropriate medications in the elderly

Before the CPs’ observation, 50 pXDDIs were recorded in the patient’s therapy, while after medication review and the physician examination, patients received 29 pXDDIs in pharmacotherapy. The number of pXDDIs in the pharmacotherapy decreased by 42% (Mann-Whitney’s U test; U = 12,000; *p* < 0.041). The number of pXDDIs for which the CP recommended drug discontinuation are shown in the Table 2.

**Table 2:** The number of pXDDIs, for which the CP recommended drug discontinuation

pXDDIs	N prior	N after	pXDDIs	N prior	N after
Amiodarone-torsemide	1	0	*ipratropium / fenoterol-tiotropium*	*3*	*2*
Amiodarone-clozapine	1	0	*quetiapine-amiodarone*	*1*	*0*
Amiodarone-warfarin	1	0	*quetiapine -domperidone*	*2*	*1*
Etoricoxib-meloxicam	1	0	*quetiapine -escitalopram*	*7*	*3*
Haloperidol-sulpiride	2	1	*quetiapine-metoclopramide*	*3*	*2*
Haloperidol-metoclopramide	1	0	*quetiapine-sulpiride*	*2*	*1*
Cholecalciferol-calcitriol	3	0	*metoclopramide-trimetazidine*	*2*	*1*
Ipratropium/fenoterol-Olanzapine	2	1	*sulpiride-risperidone*	*1*	*0*
Not accepted pXDDIs	N prior	N after	pXDDIs	N prior	N after
Amisulpride-sulpiride	1	1	*quetiapine-budenoside / formoterol*	*1*	*1*
Desloratadine-mirtazapine	1	1	*quetiapine-domperidone*	*2*	*1*
Escitalopram-sotalol	1	1	*quetiapine-escitalopram*	*7*	*3*
Haloperidol-sulpiride	2	1	*quetiapine-haloperidol*	*3*	*3*
Haloperidol- ipratropium/phenoterol	1	1	*quetiapine-metoclopramide*	*3*	*2*
Haloperidol ipratropium/phenoterol-	1	1	*quetiapine-sulpiride*	*2*	*1*
Olanzapine- ipratropium/phenoterol	2	1	*quetiapine-trazodone*	*1*	*1*
Quetiapine-tiotropium	1	1	*quetiapine-tiotropium*	*1*	*1*
Carbamazepine-clozapine	3	2	*metoclopramide-trimetazidine*	*2*	*1*
Carvedilol- budenoside/formoterol	2	2	*olanzapine-thyrotropium*	*1*	*1*
Quetiapine- ipratropium/phenoterol	1	1	*sulpiride-trimetazidine*	*1*	*1*

The pXDDIs were clinically relevant in six patients (*n* = 6; 6.6%), all of whom experienced a QT interval prolongation due to pXDDIs. In one patient, the QT prolongation occurred because of a pXDDI between haloperidol and quetiapine (a pharmacodynamic pXDDI), in three patients due to a pXDDI of escitalopram and quetiapine (a pharmacodynamic pXDDI) and in two patients due to a pXDDI of domperidone and quetiapine (a pharmacodynamic pXDDI). These results show that 12% of pXDDIs were clinically expressed.

**Fig. 2:**
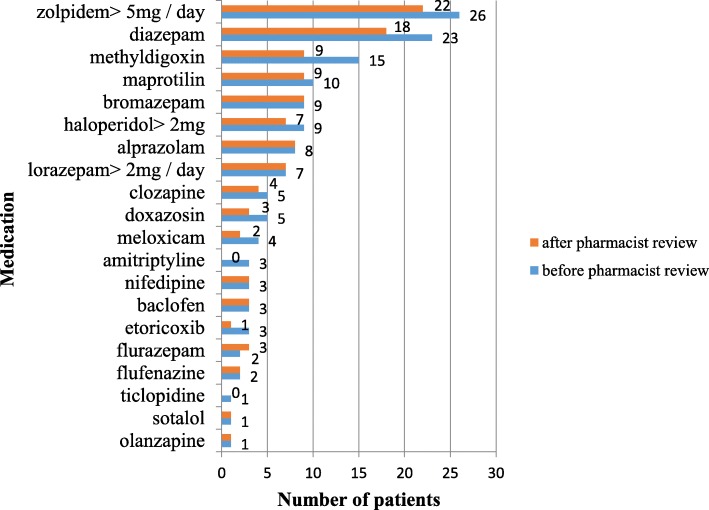
Number of patients with PIMs before clinical pharmacist review and after review according to the PRISCUS List

The pXDDI of escitalopram and quetiapine was clinically expressed in 42.9% of the cases (the number of pXDDIs of escitalopram and quetiapine was seven) and the pXDDI of domperidone and quetiapine in 100% of the cases (the number patients with this pXDDIs was two). The CPs suggested drug discontinuation in all 6 clinically important pXDDIs and 50% of the suggestions were accepted by the GPs. Prior to the CP’s review, patients received 140 PIMs in total (12.5% of total medications), which was reduced to 112 PIMs (10.0% of total medications) after the review, which is a decrease of 28 (20%) (Mann-Whitney U test; U = 87,500; *p* = 0.069). All PIMs with their numbers before and after the review are shown in the Fig. 2.

### Linear regression model results for the occurrence of potential X DDIs

In the model for pXDDIs, a statistically significant model p (χ2 = 37,612, df = 4, *p* < 0.001) was obtained by logistic regression (Table 3). The number of pXDDIs was statistically influenced by the accepted CPs’ interventions and the total number of prescribed medications (*p* < 0.005). Accepted CPs’ recommendations led to a reduction in the number of pXDDIs.

**Table 3:** Linear regression model results for the occurance of potential X DDIs

Independent variable	β value	*P* value
Clinical pharmacist acceptance	−1.955	0.003
Age	0.024	0.546
Total number of medicines	−0.442	< 0.001
Gender	0.253	0.686

### Adherence to heart failure treatment guidelines

Adherence to treatment guidelines was checked in patients with a heart failure diagnosis in their charts. 36 patients were diagnosed with heart failure (Table 4). In nine cases (25.0%), the CP’s recommendations were accepted by GPs, which means that treatment guidelines adherence was improved through accepted CP’s recommendations.

**Table 4:** Adherence to heart failure treatment guidelines (ESC Guidelines), GPs=general practitioners

Case number	Treatment guidelines issue	Clinical pharmacist recommendations	GPs acceptance (YES/NO)
**1.**	**Methyldigoxin treatment**	**Methyldigoxin discontinuation and perindopril initiation**	**YES**
2.	Methyldigoxin treatment	Methyldigoxin discontinuation and enalapril initiation	NO
**3.**	**Methyldigoxin treatment + β-blocker +ACE inhibitor**	**Methyldigoxin treatment discontinuation**	**YES**
**4.**	**Methyldigoxin treatment +ACE inhibitor**	**Methyldigoxin treatment discontinuation + β-blocker initiation**	**YES**
5.	Methyldigoxin treatment	β-blocker initiation and ACE inhibitor initiation	NO
**6, 7, 8, 9, 10.**	**Methyldigoxin treatment + β-blocker**	**Methyldigoxin treatment discontinuation**	**YES, NO, YES, NO, YES**
11.	Methyldigoxin treatment +ACE inhibitor	Methyldigoxin treatment discontinuation + β-blocker initiation	NO
**12.**	**1.25 mg bisoprolol daily**	**Drug adjustment (2,5 mg daily)**	**YES**
13, 14, 15.	ACE inhibitor treatment	β-blocker adding	NO, NO, NO
16, 17, 18.	ACE inhibitor treatment with verapamil	Verapamil discontinuation and selective β-blocker initiation	NO, NO, NO
19.	Inappropriate dosing of ACE inhibitor and β-blocker	Reduce the ACE inhibitor dose and increase the dose of the β-blocker	NO
**20.**	**Nonselective β-blocker**	**Switching to bisoprolol**	**NO, YES**
**21.**	**Amlodipine treatment**	**Amlodipine discontinuation and β-blocker (bisoprolol) initiation**	**YES**

Interventions where CP's recommendations were accepted are presented in a bold form

## Discussion

The results of this study show that a medication review service provided by CPs in primary care had a significant impact on improving the quality of pharmacotherapy, measured as the reduction in the total number of medications, PIMs and pXDDIs, and improved treatment guidelines adherence. Results show that GPs accepted almost half of the suggested interventions, which is in line with a study from a Belgian hospital (56.6%) but not with a study in a Slovenian psychiatric hospital (88.0%), which may be explained by a different work environment [20, 21]. The results also show that the main role of the CP is not only medication reduction, but pharmacotherapy optimization (e.g. better treatment guidelines adherence), which can also lead to more medications in some patients (two patients in our study). In a study in the U.S., the researchers found that only 7% of the CPs’ interventions were connected with drug discontinuation [10]. In our study, drug discontinuation represented only 13.5% of the interventions, drug initiation 2.3%, and other interventions represented almost 85%. These results confirmed that the total number of medications decreased significantly after a CP’s intervention, which had previously been already discussed in some trials [6, 9, 10]. In addition, a systematic review and meta-analysis published in 2014 confirmed our positive results. Pharmacist interventions usually involved medication reviews (86.8%), with or without other activities delivered collaboratively with the GP (family physician) [22]. There are many studies describing the collaboration between GPs and CPs within a community pharmacy setting [23]. Although our study setting was a primary care community setting, the results were also positive. This study was funded by the ZZZS that established this work environment within the Slovenian primary care community settings before. The researchers did not have an impact on the study environment. Although this setting use in this study was supported by payer, in future, existing community pharmacy network in collaborative care with primary care should be studied, so that a broader scope of evidence will be also available in future on this same service in Slovenia.

One of the most important findings of this study is that CPs’ interventions significantly reduced the total number of medications and pXDDIs. Although the potential reduction of pXDDIs as a result of involving CPs have been described elsewhere, [6, 9, 10], our study extends the findings to at a primary care setting. The most frequent pXDDIs were between quetiapine and escitalopram which the CP’s interventions reduced from 6 to 3 cases. Quetiapine was a medication which was involved in most of pXDDIs and was discontinued in many cases as suggested by the CP. These results are in line with the clinical guidelines for antipsychotic use and insomnia treatment, according to which quetiapine is not a first line treatment, especially for insomnia, because the evidence is very weak [24–26]. Therefore the CP’s suggestions about quetiapine discontinuation were evidence-based [27, 28]. These results suggest that CPs can support GPs in treating insomnia and managing the use of antidepressants to avoid important pXDDIs. 6.6% of patients experienced clinically important pXDDIs as an adverse event and the CP suggested drug discontinuation to avoid pXDDIs. This means that a CP can also reduce clinically important pXDDIs, which is also in line with previous results [6, 9, 10]. Our regression model also showed that the number of pXDDIs was statistically influenced by the involvement of the CP and the total number of prescribed medications (< 0.005), which means that the CP’s interventions had an impact on the total number of pXDDIs (high β value) which is in line with previous results [6].

The next important finding is connected with a lower number of PIMs after CPs’ interventions. These results show that PIMs were very frequent in this study population, especially PIMs in psychotropics. The CPs’ interventions reduced PIMs by 20%, although non-significantly, which can be explained by the small sample size used in this study. Although the results were not significant, CPs’ interventions led to a reduction in several important PIMs (e.g. amitriptyline). CP’s interventions led to the discontinuation of several drugs (e.g. haloperidol, methyldigoxin, amitriptyline and different benzodiazepines), which is in line with the PRISCUS list [18].

The last important finding of this paper is connected with better treatment guidelines adherence, which is particulary important for better clinical outcomes. The CP suggested pregabalin in a case of neuropathic pain and the sedative antidepressant mirtazapine instead of amitriptyline, which is also supported by clinical guidelines [27]. Methyldigoxin was often replaced by beta-blockers and/or ACE inhibitors in patients with chronic heart failure, which is also in line with clinical guidelines [19]. These results show that patients, after the CP’s interventions, had fewer important PIMs and were more likely to use appropriate alternatives, which confirmed the important role CPs can play in reducing PIMs in a primary care setting. In this study, only chronic heart failure treatment guidelines adherence was examined. Interestingly, inconsistencies with the treatment guidelines were found in 58.3% of all patients with heart failure, especially regarding the lack of the most advisable treatment strategy, which can have an impact on final patient-survival (e.g. β-blockers and ACE-inhibitors or ARBs) [19]. These results show that treatments followed guidelines more appropriately if the recommendations by the CPs’ were accepted by the GP. These results are not in line with a small sized Slovenian study, where hospital CPs’ interventions were included. In this small-size randomized controlled trial (RCT) (intervention, *n* = 26; control, *n* = 25) pharmacist intervention significantly reduced the number of patients with clinically relevant DDIs, but not clinical endpoints 6 months from discharge (re-hospitalization or death) (10 vs. 7; *p* = 0.74) [29]. In our study, dose adjustment was suggested in patients with chronic heart failure in many cases, which is also in line with the clinical guidelines [19]. These results showed that a collaborative care approach including a CP is beneficial for patients with chronic heart failure, although more studies with bigger sample sizes are needed to confirm these results.

In addition, a clinical pharmacy service is often an cost-effectivenes approach in the medication management process in elderly patients, as was calculated by Lee and co-authors at a VA medical center. Our results were also economically positive with return on investment 5:1, which was calculated in MPharm thesis already (not included in the manuscript), which confirms a cost-effectivenes of these interventions in real clinical practice. However, these economic outcomes are interesting, there are many limitations predominantly because of a lack of Slovenian costs [30–32]. These results together with our previous results show that payers in Slovenia should stimulate clinical pharmacy service implementation in pharmacotherapy optimization process in elderly patients on polypharmacy (e.g. better clinical outcomes and lower costs) [31, 32].

Lastly, this study also has many important limitations, which should be addressed. The most important limitation is an absence of a control group as well as of humanistic and/or clinical outcome measures. (no between group difference and no treatment outcomes differences). These limitations are due to the design of the *Pharmacist Consultant* pilot trial, in which no control group or patient-specific measurements (e.g. questionnaires) were planned and it was impossible to obtain the data retrospectively. GPs also referred patients to CPs without strict protocol, which can be a source of selection bias. We also didn’t have any data about patient refusals, which can have an impact on the sample size. In addition, a retrospective cohort design has many other important limitations, which should be addressed (e.g. selection bias, attrition bias and missing data). The second limitation is that the results of pDDIs may also depend on the Lexicomp® software version, as the standards used are frequently updated [16, 33]. The third important limitation is that we included only X-type pDDIs, although some authors argue that the pDDIs of category C and D are also clinically relevant. This means some potentially important interventions were not included in the analysis to minimize the number of non-important pDDIs, so the effects of this study then might be lower than anticipated. We should also acknowledge the absence of socio-economic variables (e.g. no. school years, employment situation, income) collected which prevented to assess health equity issues of the intervention provided, as recommended for the evaluation of health care interventions, namely to compare differences in expected outcomes between low and high socio-economic subsets. A public health intervention (included those delivered by pharmacists) should ideally produce greater improvements in more deprived socio-economic patients to ensure health equity. This requires further investigation in future research.

However, this observational study still offers new insights into the merits of a collaborative care model including GPs, expands the knowledge of collaborative model implementation in Central Europe and is one of the first papers describing elderly patients with polypharmacy in a collaborative primary care setting.

## Conclusions

A collaborative care model including CPs undertaking a medication review service in primary care led to fewer pXDDIs per patient, fewer total medications per patient, fewer PIMs per patient, and better treatment guidelines adherence in patients aged 65 years and older on 10 or more medications in Slovenia. Although these positive results, a further research considering an experimental randomized or quasi-randomized controlled design is necessary.

## Abbreviations

ACE: Angiotensin-converting enzyme
ARBs: Angiotensin II Receptor Blockers
CP: Clinical pharmacist
ESC: European Society of Cardiology
GPs: General practitioners
pDDIs: Potential drug-drug interactions
PIMs: Potentially inappropriate medications in the elderly
pXDDIs: Potential X-type DDIs
RCT: Randomized controlled trial
SD: Standard deviation
ZZZS: Health Insurance Institute of Slovenia
